# Time to first recurrent thrombosis in adults with thrombotic antiphospholipid syndrome: a registry-based retrospective cohort study

**DOI:** 10.1007/s10067-026-08372-9

**Published:** 2026-09-08

**Authors:** María Clara Escobar-Millán, Simón Correa-Sierra, Sebastian Hoyos-Gutierrez, Laura Lopez-Correa, Carolina Ochoa-Gallego, Diana Rocío Gil Calderón, Juan Camilo Díaz-Coronado, Diego Fernando Rojas-Gualdrón

**Affiliations:** 1 Facultad de Ciencias de La Salud, Universidad CES, Calle 10ª # 22-04 Bloque C Piso 2 Facultad de Ciencias de la Salud, Medellín, Colombia; 2 ARTMEDICA IPS, Medellín, Colombia; 3 Hospital Universitario Mayor-MEDERI – Universidad El Rosario, Bogotá, Colombia

**Keywords:** Autoimmune diseases, Antiphospholipid syndrome, Recurrence, Thrombosis

## Abstract

**Objective:**

To estimate time to first recurrent thrombosis and to explore the association between recurrent thrombosis and clinical, laboratory/immunological, and treatment-related factors among Colombian adults with thrombotic antiphospholipid syndrome (APS) enrolled in the ARTMEDICA autoimmune diseases program (2013–2024).

**Methods:**

We conducted a registry-based retrospective follow-up study of patients with thrombotic APS. Patients were followed from the index thrombotic event to the first recurrent arterial or venous thrombosis. Time-to-event analyses used interval-censored parametric survival regression with a log-logistic distribution. To explore factors associated with recurrence, we also applied a matched nested case–control design using conditional logistic regression.

**Results:**

We included 355 patients (mean follow-up 6.6 years), 71.0% female, median age 47 years (IQR 32–60). Primary APS accounted for 83.4% of cases, and the index event was arterial in 72.1%. During follow-up, 130 patients experienced a first thrombotic recurrence: 70.8% venous. The 10-year probability of first recurrence was 42% (95% CI 28.4–55.5%), with 20% (95% CI 10.5–29.1%) during the first 2 years. In the nested case–control, recurrent thrombosis was significantly associated with the LA + /aCL + /antiβ2GPI − profile (OR 4.8; *p* = 0.044), male sex (OR 1.8; *p* = 0.038), prior gestational death (OR 2.4; *p* = 0.008), and ANA positivity (OR 2.4; *p* = 0.015).

**Conclusions:**

The risk of recurrent thrombosis was highest during the first 2 years after the index thrombotic event, supporting an early vulnerable phase and underscoring the need for closer clinical surveillance and secondary prevention. Limitations of the study include its retrospective design, the lack of standardized anticoagulation monitoring and residual confounding.

**Table:** 

**Key Points** *• Thrombotic APS carries a high early recurrence burden despite anticoagulation, particularly during the first 2 years after the index event.* *• Early identification of high-risk patients and intensive surveillance may be critical to prevent recurrent thrombosis.*

## Introduction

Antiphospholipid syndrome (APS) is a systemic autoimmune disease characterized by obstetric morbidity, arterial and/or venous thrombotic events, and/or microvascular manifestations in the presence of persistent antiphospholipid antibodies: lupus anticoagulant, anticardiolipin antibodies, and anti‑β2‑glycoprotein I [1]. Despite some characteristics commonly observed among APS patients, the APS ACTION (2010–2019) registry highlighted the heterogeneity of aPL-related clinical manifestations and laboratory profiles, overall and among patients with APS [2]. APS is a condition with significant morbidity and mortality with a high burden attributable to thrombosis [3, 4].

Thrombotic APS is the most frequent and life-threatening presentation of APS, particularly for patients with confirmed recurrences or at high risk [5]. Recurrent thrombosis is defined as a new confirmed arterial or venous thrombotic event occurring after the index event in the absence of another provoking factor [6]. Despite anticoagulation, recurrences are not uncommon with estimated pooled rates of 5.4% and 19.7% following initial venous and arterial events, respectively [7], and real‑world series reporting rates as high as 46% [8]. These recurrences are associated with even higher morbidity, mortality, and damage [9], with catastrophic APS playing a significant role [10].

Clinical, laboratory, and treatment‑related factors have been linked to recurrent thrombosis in APS [11, 12]. However, due to the heterogeneity of clinical manifestations and laboratory profiles, prediction of recurrence is challenging [13], and studies describing its trajectory by relevant patient characteristics are scarce, particularly among Latin American populations. Therefore, in this study of Colombian adults with thrombotic APS enrolled in the ARTMEDICA program for autoimmune diseases between 2013 and 2024, our primary objective was to estimate the time to first recurrent thrombosis by relevant characteristics of the index event. As a secondary objective, we explored the association of recurrent thrombosis with clinical manifestations, laboratory and immunological findings, risk factors, and treatment at the most recent medical assessment.

## Materials and methods

This study was approved by the Institutional Review Board of CES University (Ae-1298, 2024) and conducted in accordance with the Council for International Organizations of Medical Sciences (CIOMS) ethical principles and guidelines on the use or clinical records for health research [14]. All patients provided informed consent at program entry. This report follows the STROBE recommendations for observational studies [15].

### Study design and setting

We conducted a registry-based retrospective follow-up study of patients with thrombotic APS, who were followed from the index thrombotic event to the first thrombotic recurrence, death or the last available medical assessment. Patients admitted to ARTMEDICA’s rheumatology service between 2013 and 2024 were considered eligible; data collection was carried out during 2025. ARTMEDICA is a specialized care center for autoimmune diseases in Colombia, with presence in twelve cities.

### Participants

Potentially eligible patients were identified by filtering institutional datasets using ICD‑10 code D68.61 (APS). Medical records of potentially eligible patients were reviewed to verify inclusion criteria: (1) age ≥ 18 years; (2) diagnosis of APS confirmed by the rheumatologist at program enrolment, and (3) confirmed history of the index arterial or venous thrombotic event. Patients with insufficient information in the medical record to reconstruct the history of thrombotic events and those who did not provide informed consent were excluded. Follow‑up was conducted by sequential review of medical records.

### Variables and data sources

The primary outcome was time from the index thrombotic event to the first recurrent arterial or venous thrombosis. Additional variables included demographics and clinical characteristics at the time of the index event; clinical manifestations and risk factors recorded at the most recent assessment, as well as immunological and other laboratory findings, pharmacologic treatment, and variables from the adjusted Global APS Score (aGAPSS) [16]. All variables were extracted from structured clinical and laboratory records documented by attending rheumatologists as part of routine care within the ARTMEDICA follow‑up program.

### Bias

To reduce misclassification and information bias, clinical and administrative records were linked using unique patient registration codes, and data extraction was performed by five third-year residents of internal medicine, under supervision of senior clinical data specialists. Multivariate regression models matching by time to event or censoring and anticoagulation were used to control confounding factors. Data regarding anticoagulation adherence or additional interventions prior to the initial follow-up in ARTMEDICA rheumatology program were not available.

### Study size

The study included 355 patients who contributed 2333.56 patient‑years of follow‑up, with a mean follow-up 6.6 years. Of these, 228 patients were right‑censored, 28 were left‑censored, and 99 were interval‑censored with a median interval width of 2.5 years. Post hoc analysis of the final interval‑censored loglogistic survival regression model showed a precision of ± 2.6 years (relative precision 20.3%) for the estimated median time to first recurrent thrombotic event; ± 6.5% (relative precision, 10.0%) for 5‑year recurrence‑free survival; and ± 7.1% (relative precision, 13.2%) for 10‑year recurrence‑free survival probability.

### Statistical methods

Descriptive analysis of patient characteristics at the time of the index event was performed with frequencies and percentages for categorical variables and with median and interquartile range (IQR) for quantitative variables.

### Time to first recurrent thrombosis by relevant characteristics of the index event

To model time to first recurrence, we fitted interval‑censored parametric survival regression models and evaluated several distributions; the loglogistic distribution, an accelerated failure time (AFT) model, provided the best fit by Akaike (AIC) and Bayesian information criteria (BIC); interval-censoring was applied to patients where the specific date of recurrence was not observed. For these patients, the interval was defined using the date of the last clinic visit at which the patient was still recurrence-free and the date of the next visit at which the recurrence was identified. The width of these intervals varied between patients, depending on follow-up schedule, patient compliance, and access-related administrative factors. Variables with *p* < 0.25 in simple regression models were considered for the multivariable model, and the final model was selected based on best fit statistics (AIC and BIC); adjusted time ratios (aTR) with 95%CI are presented. Median time to first thrombotic recurrence with 95% confidence intervals (95% CI) and the survival and hazard functions were obtained as marginal estimates from the final model and plotted through 10 years of follow‑up.

### Association of the recurrent thrombosis with patient characteristics at the most recent medical follow-up

We used a matched nested case–control design to perform exploratory analyses of association while properly accounting for time to event, censoring and confounding, following the recommendations of Partlett et al. [17]. Besides time to event or censoring, specific anticoagulation type (warfarin, DOAC, etc.) was the only additional matching factor as it has a relevant confounding by indication effect [18]. We defined two controls per case which were randomly selected by sampling without replacement from risk sets constructed for each specific case from data of all patients that remained recurrence-free at the time of each event. Adjusted Odds Ratios (aOR) with 95%CI were estimated by conditional logistic regression. Variables for the final model were selected following the same criteria applied to survival regression models.

### Sensitivity analyses

We performed three sensitivity analyses: (1) influence of death as a competing risk: we estimated cumulative incidence function adjusting by the effect of death as a competing risk to recurrence and compared it with the failure probabilities obtained in the main analysis, (2) influence of the survival probability distribution: we estimated and compared median survival time and cumulative failure probabilities obtained by generalized gamma and lognormal distributions, the second and third best fit distributions according to AIC and BIC, with the main analysis performed with loglogistic distribution, and (3) influence of circumstantial risk factor for thrombosis: we estimated the main analysis model on the subgroup of patients without recent surgery, recent hospitalization, major trauma, or malignancy, and compared estimations with those obtained in the full cohort.

Statistical significance was set at 0.05. Analyses were performed in Stata 16.1 (College Station, TX). All patients with data available on the history of thrombotic events were analyzed, no registry was excluded due to missing data on covariates.

## Results

### Participants

Of 380 patients confirmed for APS with a documented history of thrombosis, recurrence history could not be reconstructed for 25 patients (6.6%), who were excluded. The final analytic cohort comprised 355 patients, contributing a total of 2333.56 patient-years of follow-up (mean follow-up 6.6 years).

### Descriptive data

Baseline characteristics at the time of the index thrombotic event are summarized in Table 1. Among 355 patients, the median age at first thrombosis was 47 years (IQR, 32–60), and 71% were female. Most patients (97.5%) were of mixed ethnicity. Primary APS accounted for 83.4% of cases, secondary SLE‑related APS for 9.9%, and secondary non‑SLE APS for 6.8%; approximately half of the latter were associated with Sjögren syndrome. The index event was venous in 72.1% of patients and arterial in 25.4%; only 2.5% presented with concomitant arterial and venous thrombosis. Among venous index events, deep or superficial lower-extremity vein thrombosis (42.5%) and pulmonary embolism (20.3%) accounted for 87% of cases. Among arterial events, ischemic stroke or transient ischemic attack (17.2%) and acute limb ischemia (3.1%) accounted for 80% of cases. Associated hereditary thrombophilia was documented in 5.6% of patients, with protein C or protein S deficiency (2.5%) accounting for nearly half of these abnormalities. Clinical manifestations, risk factors, and treatment at the most recent medical assessment are presented in Table 2, while laboratory and immunological findings from the same assessment are presented in Table 3.

**Table 1:** Patient characteristics at the time of the index thrombotic event

	*n*	%
Age. Median (IQR)	47	(32–60)
Sex		
Female	252	71.0
Male	103	29.0
Ethnicity		
Mixed race	346	97.5
African American	5	1.4
Indigenous	1	0.3
Other/no data	3	0.9
Type of APS		
Primary	296	83.4
Secondary, SLE-related	35	9.9
Secondary, other rheumatic diseases	24	6.8
- Sjögren disease	11	(3.1)
- Rheumatoid Arthritis	2	(0.6)
- Systemic Sclerosis	2	(0.6)
- Systemic vasculitis	1	(0.3)
- Other	8	(2.3)
Type of thrombosis		
Arterial thrombosis	90	25.4
- Central nervous system (stroke, TIA)	61	(17.2)
- Acute myocardial infarction and unstable angina	4	(1.1)
- Acute limb ischemia	11	(3.1)
- Retinal arterial occlusive disease	7	(2.0)
- Visceral infarction (renal, splenic, mesenteric)	7	(2.0)
Venous thrombosis	256	72.1
- Deep vein thrombosis or superficial vein thrombosis of lower limb	151	(42.5)
- Pulmonary embolism	72	(20.3)
- Cerebral vein thrombosis (includes retinal vein)	24	(6.8)
- Visceral vein thrombosis (renal, mesenteric, splenic, portal, or hepatic)	8	(2.3)
- No data	1	(0.3)
Arterial and venous thrombosis	9	2.5
Hereditary thrombophilia	20	5.6
- Antithrombin deficiency	2	(0.6)
- Protein C and S deficiency	9	(2.5)
- Factor V de Leiden	4	(1.1)
- Hyperhomocysteinemia > 40 µmol/L	5	(1.4)

*IQR* interquartile range, *APS* antiphospholipid syndrome, *SLE* systemic lupus erythematosus, *TIA* transient ischemic attack

**Table 2:** Clinical manifestations, risk factors, and treatment at the most recent medical assessment

	Cases		Matched controls		Non-cases	
	*n*	%	*n*	%	*n*	%
HDP (preeclampsia, eclampsia, HELLP)	14	10.8	27	10.4	24	10.7
Gestational death	36	27.7	46	17.7	36	16.0
Premature birth	12	9.2	16	6.2	15	6.7
Cardiac manifestations						
Valvulopathy (vegetation or valve thickening)	4	3.1	9	3.5	5	2.2
Chronic or acute coronary disease	9	6.9	7	2.7	2	0.9
No	117	90.0	244	93.8	218	96.9
Cutaneous manifestations						
Livedo reticularis	1	0.8	2	0.8	5	2.2
Livedo racemosa	0	0.0	4	1.5	1	0.4
Splinter hemorrhage	1	0.8	5	1.9	2	0.9
Necrosis or gangrene	1	0.8	0	0.0	0	0.0
Ulcers	4	3.1	9	3.5	4	1.8
Pseudo vasculitic lesions	1	0.8	5	1.9	3	1.3
Livedoid vasculopathy	0	0.0	0	0.0	1	0.4
No	122	93.8	235	90.4	209	92.9
Neurological manifestations						
Epilepsy	0	0.0	9	3.5	6	2.7
Cognitive alterations	2	1.5	1	0.4	2	0.9
Chronic headache	4	3.1	5	1.9	10	4.4
Transverse myelitis	0	0.0	0	0.0	2	0.9
Sensorineural hearing loss	3	2.3	5	1.9	3	1.3
Sneddon syndrome	0	0.0	0	0.0	1	0.4
No	121	93.1	240	92.3	201	89.3
Renal manifestations						
Thrombotic microangiopathy	1	0.8	3	1.2	1	0.4
Glomerular diseases (membranous nephropathy minimal change disease, FSGS)	2	1.5	6	2.3	4	1.8
No	127	97.7	251	96.5	220	97.8
Hematologic manifestations						
Thrombocytopenia (< 130.000)	14	10.8	30	11.5	25	11.1
Immune hemolytic anemia	1	0.8	5	1.9	4	1.8
Thrombotic microangiopathy	0	0.0	3	1.2	1	0.4
No	115	88.5	222	85.4	195	86.7
Catastrophic APS	4	3.1	0	0.0	0	0.0
Risk factors						
Body mass index > 30	38	29.2	87	33.5	69	30.7
High blood pressure	50	38.5	97	37.3	76	33.8
Dyslipidemia	14	10.8	51	19.6	36	16.0
Diabetes mellitus	11	8.5	21	8.1	13	5.8
Chronic kidney disease	7	5.4	22	8.5	14	6.2
Smoking	12	9.2	19	7.3	14	6.2
Atrial fibrillation	0	0.0	1	0.4	1	0.4
Combined hormonal contraceptives	3	2.3	3	1.2	8	3.6
Malignancy	5	3.8	8	3.1	9	4.0
Recent hospitalization	8	6.2	10	3.8	10	4.4
Recent surgery	7	5.4	3	1.2	6	2.7
Major trauma	1	0.8	0	0.0	1	0.4
Anticoagulants						
Warfarin	64	49.2	173	66.5	141	62.7
Low molecular weight heparin	31	23.8	42	16.2	31	13.8
Direct oral anticoagulant	31	23.8	34	13.1	23	10.2
No	4	3.1	11	4.2	30	13.3
Aspirin	25	19.2	24	9.2	41	18.2
Hydroxychloroquine	54	41.5	62	23.8	54	24.0
Statin	41	31.5	93	35.8	59	26.2
Immunosuppression	25	19.2	47	18.1	38	16.9

*HDP* hypertensive disorders of pregnancy, *HELLP* hemolysis, elevated liver enzymes, low platelet count, *APS* antiphospholipid syndrome, *FSGS* focal segmental glomerulosclerosis

**Table 3:** Laboratory and immunological findings at the most recent medical assessment

	Cases		Matched controls		Non-cases	
	*n*	%	*n*	%	*n*	%
Immunological findings						
Lupus anticoagulant						
Positive	98	75.4	181	69.6	165	73.3
Negative	21	16.2	58	22.3	46	20.4
No data	11	8.5	21	8.1	14	6.2
Anticardiolipin IgG						
Positive	74	56.9	150	57.7	127	56.4
Negative	53	40.8	106	40.8	94	41.8
No data	3	2.3	4	1.5	4	1.8
Anticardiolipin IgM						
Positive	53	40.8	96	36.9	88	39.1
Negative	74	56.9	163	62.7	134	59.6
No data	3	2.3	1	0.4	3	1.3
Antiβ2GPI IgG						
Positive	62	47.7	126	48.5	112	49.8
Negative	60	46.2	124	47.7	105	46.7
No data	8	6.2	10	3.8	8	3.6
Antiβ2GPI IgM						
Positive	27	20.8	67	25.8	71	31.6
Negative	94	72.3	182	70.0	144	64.0
No data	9	6.9	11	4.2	10	4.4
Triple positivity						
Positive	63	48.5	138	53.1	90	40.0
Negative	46	35.4	87	33.5	106	47.1
No data	21	16.2	35	13.5	29	12.9
C3						
Consumed	13	10.0	25	9.6	22	9.8
Normal	98	75.4	212	81.5	176	78.2
No data	19	14.6	23	8.8	27	12.0
C4						
Consumed	11	8.5	28	10.8	24	10.7
Normal	100	76.9	206	79.2	172	76.4
No data	19	14.6	26	10.0	29	12.9
ANA						
Negative	23	17.7	60	23.1	56	24.9
Positive	93	71.5	161	61.9	139	61.8
No data	14	10.8	39	15.0	30	13.3
anti-dsDNA						
Positive	14	10.8	16	6.2	17	7.6
Negative	93	71.5	209	80.4	178	79.1
No data	23	17.7	35	13.5	29	12.9
Anti-ENAS (Sm, Ro, La, RNP)						
Positive	21	16.2	31	11.9	28	12.4
Negative	92	70.8	206	79.2	174	77.3
No data	17	13.1	23	8.8	22	9.8
Rheumatoid factor						
Positive	4	3.1	12	4.6	10	4.4
Negative	65	50.0	115	44.2	95	42.2
No data	61	46.9	133	51.2	120	53.3
Laboratory findings, median (IQR)						
ESR	9	(5–21)	10	(6–19)	10	(6–20)
C-reactive protein	0.38	(0.13–1.08)	0.44	(0.20–1.24)	0.4	(0.16–1.1)
Hemoglobin	14.4	(13.2–15.5)	14	(13.0–15.0)	14	(13–15)
Leukocytes, × 10^3^	6.18	(5.14–7.19)	6.54	(5.46–8.30)	6.44	(5.3–7.87)
Neutrophils, × 10^3^	3.6	(2.76–4.41)	3.87	(2.87–4.77)	3.86	(2.8–4.61)
Lymphocytes, × 10^3^	1.85	(1.5–2.21)	1.93	(1.61–2.45)	1.9	(1.58–2.42)
Platelets, × 10^3^	240	(183–303)	266	(206–314)	264	(206.5–313.5)
Creatinine	0.89	(0.74–1.07)	0.85	(0.74–1.07)	0.85	(0.73–1.045)

*IgG* immunoglobulin G, *IgM* immunoglobulin M, *β2GPI* beta-2 glycoprotein I, *C3* complement component 3, *C4* complement component 4, *ANA* antinuclear antibodies, *anti-dsDNA* anti-double-stranded DNA antibodies, *ENAs* extractable nuclear antigens, *Sm* Smith antigen, *Ro* Ro/SSA antigen, *La* La/SSB antigen, *RNP* ribonucleoprotein, *ESR* erythrocyte sedimentation rate, *IQR* interquartile range

### Outcome data

**Fig. 1:**
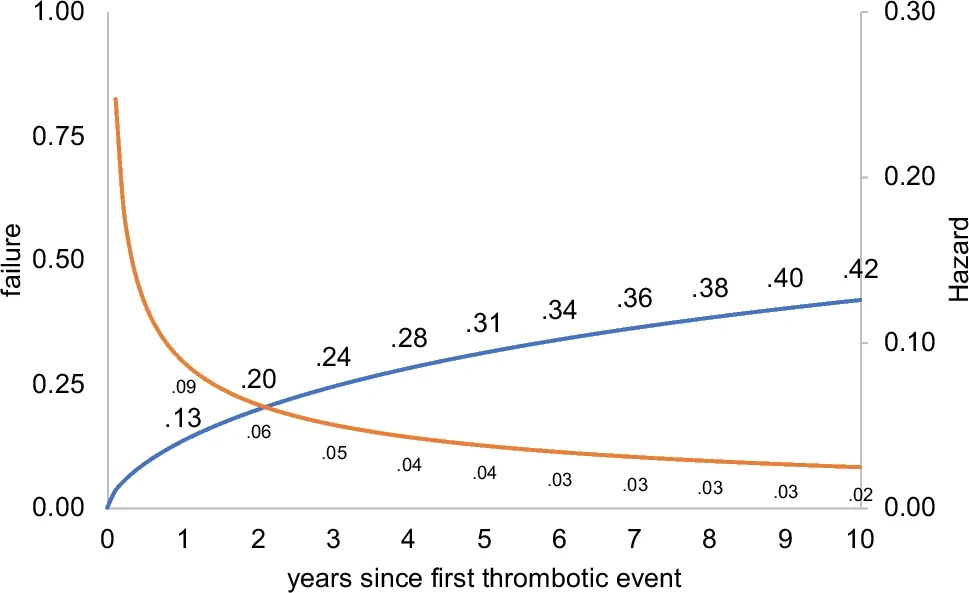
Ten-year cumulative incidence (failure) and hazard functions for first thrombotic recurrence. Failure (blue) and hazard (orange) functions for time to first recurrent thrombosis after the index thrombotic event, shown up to 10 years. The blue curve represents the cumulative probability of first recurrence (left axis), and the orange curve represents the instantaneous hazard of recurrence (right axis). Labels are rounded to two decimal places

During follow-up, 130 patients experienced their first thrombotic recurrence; 70.8% were venous and 29.2% were arterial events. Among arterial recurrences, 79% corresponded to ischemic stroke or transient ischemic attack, whereas among venous recurrences, 51% were lower-extremity deep vein thrombosis and 35% were pulmonary embolism. The 5- and 10-year probabilities of first recurrence were 31% (95% CI 18.8–43.8%) and 42% (95% CI 28.4–55.5%), respectively (Fig. 1). The first 2 years exhibited the highest risk of recurrence, as shown by the hazard function; by year 2, the cumulative probability of recurrence was 20% (95% CI 10.5–29.1%), approximately half of the 10-year estimate. The estimated median time to first recurrence was 16.2 years (95% CI 9.1–23.4).

### Time to first recurrent thrombosis by relevant characteristics of the index event

**Fig. 2:**
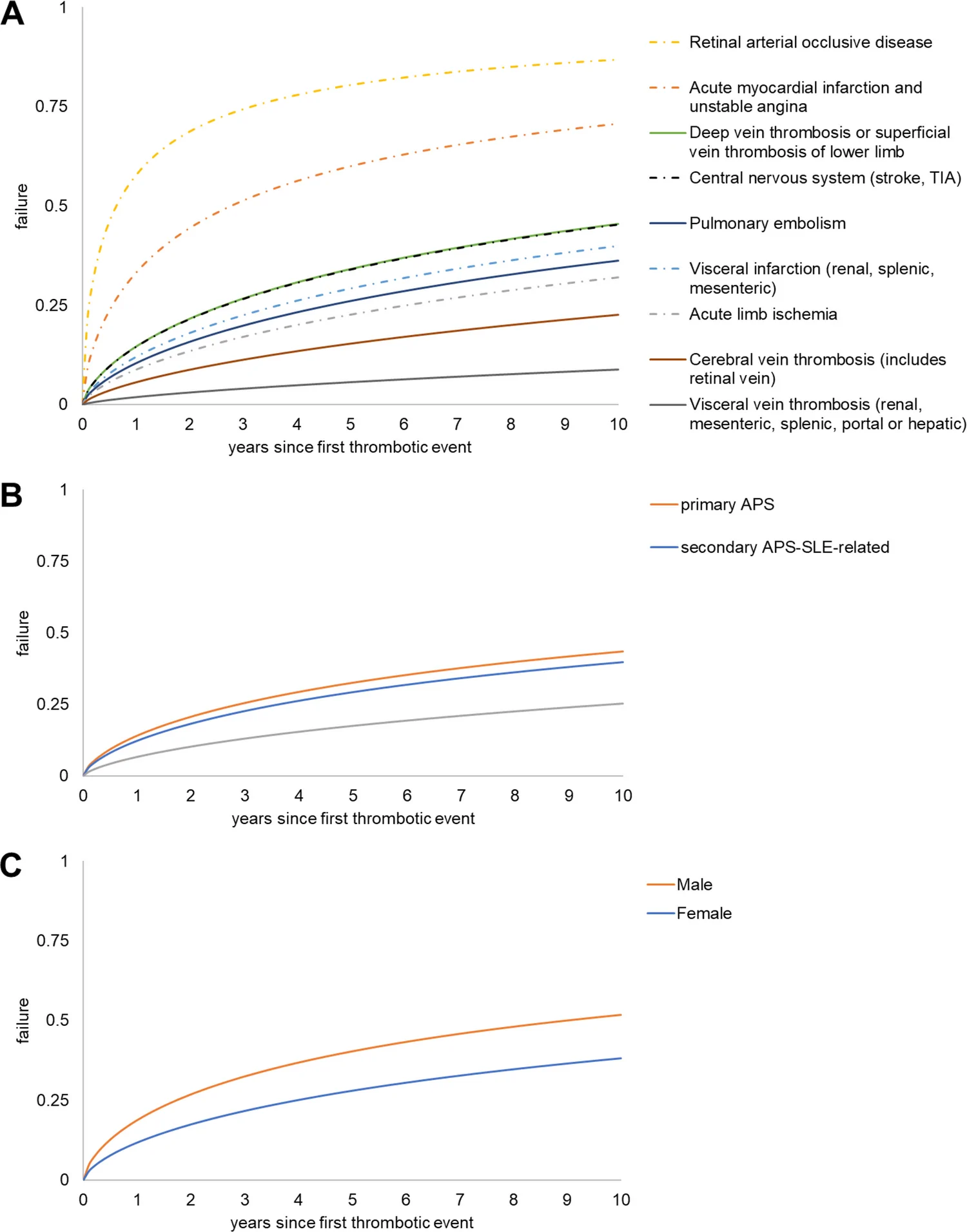
Ten-year cumulative incidence (failure function) of first thrombotic recurrence by (**A**) vascular bed of the index event, **B** APS subtype, and (**C**) sex. Failure functions (cumulative incidence) for time to first thrombotic recurrence up to 10 years after the index thrombotic event. Panels show estimates stratified by (**A**) vascular bed involved in the index event (dashed lines indicate arterial thrombosis and solid lines indicate venous thrombosis), **B** APS subtype (primary APS, secondary APS-SLE-related, and secondary APS-non-SLE-related), and (**C**) sex

There was no statistically significant difference in time to first recurrence between arterial and venous index events (aTR = 0.86; 95% CI 0.35–2.12). However, when stratified by the specific vascular bed involved (Fig. 2a), time to recurrence was significantly shorter after retinal artery occlusion (adjusted median 0.6 years; adjusted *p* = 0.006) and after acute myocardial infarction or unstable angina (adjusted median 2.8 years; adjusted *p* = 0.029).

Time to first recurrence did not differ between primary and secondary APS (aTR 1.16; 95% CI 0.42–3.20). However, when secondary APS was stratified into SLE-related versus other rheumatic disease–related APS (Fig. 2b), there was a non-significant trend toward longer time to recurrence in non-SLE secondary APS, whereas SLE-related APS and primary APS showed comparable recurrence times. In relation to sex (Fig. 2c), there was a non-significant trend toward shorter time to recurrence among men (aTR 0.59; 95% CI 0.26–1.36) who showed a higher 5-year cumulative incidence (40.3%) than women (27.9%).

### Association of the recurrent thrombosis with patient characteristics at the most recent medical follow-up

In the matched nested case–control analysis we found that at the most recent medical follow-up, several factors were more frequent in patients with recurrent thrombosis (Table 4). Among the antiphospholipid antibody profiles, the combination of LA +/aCL +/antiβ2GPI- was significantly associated with recurrence (aOR 4.8; 95% CI 1.0–21.7; *p* = 0.038). The triple positivity profile was also associated with recurrence; however, it did not reach statistical significance. Male sex (aOR 1.8; 95% CI 1.0–3.3; *p* = 0.044), prior history of gestational death (aOR 2.4; 95% CI 1.3–4.7; *p* = 0.008) and ANA positivity (aOR 2.4; 95% CI 1.2–4.8; *p* = 0.015) were also associated with higher odds of recurrence. Among the anticoagulants, DOAC use at the most recent assessment was associated with recurrence. Hydroxychloroquine use was associated with recurrence, likely reflecting cofounding by indication. Those patients with a longer time in the program showed lower odds of recurrence (aOR 0.9; 95% CI 0.8–1.0; *p* = 0.017).

**Table 4:** Adjusted association of the recurrent thrombosis with patient characteristics at the most recent medical follow-up

			OR	95%CI		*p*-value
Antiphospholipid antibody profile						
LA	aCL	antiβ2GPI				
− − −	+	−	1.0			
−	−	+	1.5	0.2	12.6	0.691
+	−	−	2.2	0.5	9.5	0.303
−	+	+	1.7	0.3	8.4	0.514
+	−	+	1.6	0.3	9.2	0.618
+	+	−	4.8	1.0	21.7	0.044
+	+	+	2.3	0.6	8.7	0.224
Male			1.8	1.0	3.3	0.038
Gestational death			2.4	1.3	4.7	0.008
Primary APS			1.7	0.8	3.6	0.136
Recent surgery			4.5	0.9	23.7	0.073
ANA positive			2.4	1.2	4.8	0.015
Anticoagulants						
Warfarin			1			
Low molecular weight heparin			1.7	0.8	3.4	0.166
Direct oral anticoagulant			2.8	1.4	5.7	0.004
Aspirin			1.9	0.8	4.7	0.136
Hydroxychloroquine			2.3	1.3	4.3	0.006
Years in the program			0.9	0.8	1.0	0.017

*OR* odds ratio, *95% CI* 95% confidence interva, *LA* lupus anticoagulant, *aCL* anticardiolipin antibodies, *antiβ2GPI* anti-beta-2 glycoprotein I antibodies, + : positive, − : negative; *APS* antiphospholipid syndrome, *ANA* antinuclear antibodies

### Sensitivity analyses

Sensitivity analysis produced failure functions estimated by competing-risk regression (cause specific hazards) and by interval-censored survival regression with generalized gamma and lognormal distribution (second and third best fit according to AIC and BIC) showed similar results to those obtained by the main analysis suggesting robustness to the influence of death as competing risks for recurrence and to differences attributable to the assumed event‑time distribution. Median survival estimates were 17.0 (95% CI 8.9–25.3) for generalized gamma and 16.4 (95% CI 9.8–23.1) with lognormal distribution, compared to a median of 16.2 years (95% CI 9.1–23.4) obtained from the main analysis. In relation to death as a competing risk, three patients died before APS recurrence, the obtained cumulative incidences were 19.1%, 28.2%, and 42.1% for 2, 5, and 10 year, compared to the cumulative estimations of 20%, 31%, and 42% from the main analysis, respectively.

The subgroup analysis performed among patients without risk factors for prothrombotic state (83.7%) produced an estimated median time to first recurrence was 17.3 years (95% CI 8.8–25.7), compared to an estimated median of 16.2 years (95% CI 9.1–23.4) obtained from all patients.

## Discussion

This study represents the largest cohort of patients with thrombotic antiphospholipid syndrome (APS) conducted in Colombia and one of the largest nationwide cohorts in Latin America to report long-term outcomes in this population. In contrast to previously available national data, largely limited to small cross-sectional studies with few recurrent events, our study provides a comprehensive evaluation of recurrence patterns and identifies independent predictors of thrombotic recurrence in a real-world setting.

Our findings confirm a substantial burden of recurrent thrombosis in APS, with 5- and 10-year recurrence probabilities of 31% and 42%, respectively, and a particularly high risk within the first 2 years following the index event. By year 2, the cumulative probability of recurrence was 20%. These results are consistent with data from the UK, where a 10-year recurrence rate of 46% has been reported, with the highest incidence observed during the early years of follow-up [19]. Similarly, a Brazilian cohort demonstrated a recurrence rate of 38.3% over a median follow-up of 7 years [20]. Together, these findings support a consistently high burden of recurrent thrombosis across diverse populations, particularly in the early phase after the initial event.

Our study suggests that the distribution of primary and secondary thrombotic APS in this Colombian population differs from that reported in large European and North American cohorts. Primary APS was the most frequent presentation in our cohort (83.4%), whereas secondary APS accounted for only 16.6%. This contrasts with data from the Euro-Phospholipid Project, in which primary APS represented 53.1% of cases [21]. Even when compared with another Colombian cohort, where 67.7% of patients were classified as having primary APS, our study demonstrates a substantially higher proportion of primary disease [22]. While true epidemiological differences cannot be excluded, the magnitude of this discrepancy suggests that structural and system-level factors may influence disease classification and reporting. Variations in referral patterns, diagnostic labeling and administrative coding practices may preferentially assign patients with coexisting autoimmune conditions to their primary rheumatic diagnosis, potentially leading to under recognition of secondary APS. This has important epidemiological implications, as misclassification may distort estimates of disease burden, recurrence risk, and treatment patterns.

The relatively older age of our cohort compared with other previously reported APS cohorts may have contributed to recurrent arterial thrombosis through mechanisms related to accelerated atherosclerosis. Although traditional cardiovascular risk factors were similar between patients with and without recurrence, the registry did not have the information about the cumulative burden, duration, severity, or degree of control. Therefore, it cannot be excluded residual confounding due to underlying atherosclerotic disease, especially in coronary, retinal, and cerebrovascular recurrent events and suggest the interaction between both diseases.

Consistent with findings from other cohorts, including the Euro-Phospholipid Project, the most common thrombotic events in our study were ischemic stroke, transient ischemic attack, deep vein thrombosis, and pulmonary embolism.

In our cohort, there was no statistically significant difference in time to first recurrence between primary and secondary APS. However, data from a single-center study by Abu-Zeinah [23] reported a significantly higher risk of thrombotic recurrence among patients with concomitant autoimmune diseases compared with those with primary APS (HR 4.93; 95% CI 1.7–14.3; *p* = 0.04). In contrast, our analysis showed a non-significant trend toward a longer time to recurrence in patients with non-SLE secondary APS, whereas those with SLE-associated APS exhibited recurrence patterns more similar to primary APS. Although this observation did not reach statistical significance, it may suggest a relatively higher early recurrence risk in SLE-associated APS. The lack of statistical significance is likely attributable to limited statistical power, given the relatively small size of the secondary APS subgroup (*n* = 59), particularly the SLE subset (*n* = 35).

Sex-related differences were also observed, with men exhibiting shorter time to recurrence and higher cumulative incidence. This finding is consistent with European data showing higher recurrence rates in men, often associated with a higher burden of cardiovascular risk factors [24]. However, in our cohort, traditional cardiovascular risk factors were similarly distributed between sexes. This discrepancy suggests that factors beyond conventional cardiovascular risk profiles may contribute to sex-related differences in APS, including endothelial dysfunction, immune-mediated mechanisms, or differential treatment response.

In exploratory analyses, male sex, history of gestational death, and ANA positivity were independently associated with recurrent thrombosis. In our cohort, ANA positivity remained independently associated with recurrent thrombosis after multivariable adjustment, suggesting that even in patients classified as having primary APS, ANA positivity may be associated with a more severe thrombotic phenotype. This is consistent with prior reports indicating that ANA-positive primary APS patients exhibit higher rates of triple antiphospholipid antibody positivity and recurrent events [25]. In contrast, findings from the APS ACTION Registry did not confirm this association [26], possibly reflecting differences in ANA testing methods, thresholds for positivity, or population-specific factors. These findings raise the possibility that ANA positivity may serve as a surrogate marker of a broader autoimmune milieu, warranting closer clinical surveillance and potentially more aggressive risk stratification.

Regarding the autoantibody profile, lupus anticoagulant (LA) positivity, particularly when combined with other antiphospholipid antibodies, emerged as a key predictor of recurrence. Interestingly, the estimated risk appeared higher in patients with LA + anticardiolipin positivity than in those with triple positivity; however, this finding should be interpreted cautiously as the relatively small number of patients in this subgroup resulted in a wide confidence interval, indicating limited precision of the effect estimate. Nevertheless, our results are consistent with international evidence highlighting the central role of LA in thrombotic risk stratification, regardless of the total antibody burden [19].

Finally, differences in treatment patterns at follow-up were observed between patients with and without recurrence, particularly in anticoagulation strategies. However, we did not have enough information to determine the burden of recurrence attributable to treatment failure. Patients with recurrent thrombosis showed a higher use of LMWH and DOACs, likely reflecting treatment adjustments following recurrent events. These findings underscore the complexity of long-term management in APS and the potential influence of confounding by indication in observational settings.

Several limitations of this study warrant consideration. First, its retrospective design, based on medical record review, may be subject to information bias. In addition, some patients experienced both the index thrombotic event and subsequent recurrence prior to enrolment, which may have introduced imprecision in the timing of exposures and outcomes, potentially affecting time-to-event estimates.

Second, no formal prior sample size calculation was performed because this was a registry-based study that included all eligible cases available in the institutional database during the study period; however, we present post hoc estimations of precision for key outcome statistics. Third, the study population was derived from a referral center, which may overrepresent patients with more severe or complex diseases, thereby introducing potential selection bias and limiting generalizability of our findings to patients managed in community practices, general hospitals or other non-specialized healthcare settings. Furthermore, variability in the quality of clinical documentation may have affected data capture; however, this limitation was partially mitigated using a structured follow-up program, which enabled data supplementation through multiple clinical encounters.

Fourth, APS classification in this study was based on the treating rheumatologist’s clinical judgment, generally aligned with the 2006 revised Sapporo criteria [27], rather than the more recent 2023 ACR/EULAR classification criteria [1], which may limit comparability with contemporary cohorts. It is important to note that the 2023 ACR/EULAR criteria are relatively recent and, despite being increasingly discussed in high-impact scientific literature, their adoption in routine clinical practice remains variable and the use of the 2006 criteria in this cohort likely reflects prevailing clinical practice during the study period, while also highlighting the transitional phase in APS classification frameworks. However, due to differences in sensitivity and specificity, reclassification using the new criteria could potentially change the composition of the cohort and its influence on recurrence-free survival trajectories must be analyzed in future studies.

Fifth, although multivariable models and matching strategies were employed to adjust for confounding, residual confounding inherent to observational studies cannot be excluded, particularly for variables not systematically captured in routine clinical practice, such as time in therapeutic range (TTR) or the INR measured immediately before or at the time of recurrent thrombosis, and treatment adherence. Because many patients entered follow-up at the specialized rheumatology center after their index or recurrent event, therefore we were unable to distinguish recurrent events occurring despite adequate anticoagulation from those potentially related to adherence issues. The inability to account for TTR and serial INR measurements may have influenced the estimates of recurrence risk. Additionally, systematic screening for hereditary thrombophilias was not routinely performed due to the real-world retrospective design of the study, and documentation was only available for the 5.6% of patients who tested positive. Sensitivity analyses excluding this subgroup showed virtually unchanged results. Transient factors such as surgery, trauma, or new comorbidities can further increase hypercoagulability in APS patients. Since one of our main goals was to reflect everyday clinical practice, where these complexities are common, we also conducted sensitivity analyses excluding patients with such triggers. The results remained virtually unchanged. The relatively low use of aspirin co-therapy among patients with arterial events may have also contributed to the lack of significant associations between treatment type and time to first recurrence. Finally, the registry did not systematically annexed individual lupus anticoagulant assay results (e.g., dRVVT and aPTT) or quantitative antiphospholipid antibody titers, inherent to real-world data, which may have introduced variability in the assessment of immunological parameters. Despite these limitations, our findings appear robust. The large sample size, extended follow-up, and data derived from a structured care program strengthen the internal validity of the study, and the consistency of our results with international cohorts supports their external relevance. These data provide valuable insights into thrombotic recurrence patterns in APS within a real-world Latin American setting.

Importantly, our results are consistent with findings from other cohorts in Latin America and internationally, which supports their external validity and suggests that these observations may be applicable to similar populations in other Latin American settings and among patients receiving specialized care for APS. Nevertheless, extrapolation to populations with different healthcare systems, genetic backgrounds, or disease phenotypes should be undertaken with caution.

In conclusion, in this Colombian cohort of patients with APS, the time to thrombotic recurrence was similar to what has been reported in previous studies, with a recurrence probability of 31% at 5 years and 42% at 10 years. The risk of recurrence is notably higher in the first 2 years after the first thrombotic event. These findings support closer clinical surveillance, including more frequent follow-up appointments during the first two years after the index thrombotic event, particularly in patients with high-risk antibody profiles (LA positivity) or ANA positivity, together with optimization of secondary thromboprophylaxis.

## Data Availability

The data supporting this study's findings are available from the corresponding author upon reasonable request.
